# Association between dietary sodium, potassium, and the sodium-to-potassium ratio and mortality: A 10-year analysis

**DOI:** 10.3389/fnut.2022.1053585

**Published:** 2022-11-11

**Authors:** Yu-Jin Kwon, Hye Sun Lee, Goeun Park, Ji-Won Lee

**Affiliations:** ^1^Department of Family Medicine, Yonsei University College of Medicine, Yongin Severance Hospital, Seoul, Gyeonggi, South Korea; ^2^Biostatistics Collaboration Unit, Department of Research Affairs, Yonsei University College of Medicine, Seoul, South Korea; ^3^Biomedical Statistics Center, Samsung Medical Center, Research Institute for Future Medicine, Seoul, South Korea; ^4^Department of Family Medicine, Severance Hospital, Yonsei University College of Medicine, Seoul, South Korea

**Keywords:** sodium, potassium, sodium-to-potassium ratio, mortality, cohort study

## Abstract

There is inconclusive evidence of the association between dietary sodium, potassium, and the sodium-to-potassium ratio and all-cause and cardiovascular disease mortality. To investigate the association between dietary sodium, potassium, and the sodium-to-potassium ratio and all-cause and cardiovascular disease mortality risks. Data from 143,050 adult participants were analyzed from prospective 10-year community-based cohort analysis. Dietary sodium, potassium, and the sodium-to-potassium ratio at baseline were assessed by a food frequency questionnaire. In Cox proportional hazards regression models, the association between dietary sodium, potassium, and their ratio and all-cause and cardiovascular disease mortality was estimated using hazard ratios and 95% confidence intervals, and their predictive ability as mortality predictors was evaluated using Harrell’s c-index. During the mean (range) 10.1 (0.2–15.9) years of follow-up, 5,436 participants died, of whom 985 died of cardiovascular causes. After adjustment for age, sex, body mass index, alcohol intake, smoking, regular exercise, total calorie intake, dyslipidemia, hypertension, diabetes, chronic kidney diseases (CKDs), and potassium or sodium intake, respectively, sodium intake was unassociated with all-cause mortality whereas potassium intake was significantly associated inversely with all-cause (Quintile-5 vs. Quintile-1, hazard ratio, 95% confidence interval, 1.09, 0.97–1.22, and 0.79, 0.69–0.91, respectively). The sodium-to-potassium ratio was not significantly associated with all-cause mortality in the adjusted model, and similar trends were observed for cardiovascular disease mortality.

## Introduction

Sodium (Na) and potassium (K) are essential nutrients that serve many physiological functions, including the maintenance of plasma volume, osmolality, and resting membrane potential (1, 2). Despite their opposing action, Na and K are intricately related to blood pressure (BP), kidney function, and cardiovascular health (3). The relationship between Na intake and BP was ascertained in large trials, including INTERSALT (4), PURE (5), and Dietary Approaches to Stop Hypertension-Sodium (6). Clinical trials and epidemiological studies have shown that high sodium intake is associated with an increased risk of cardiovascular diseases (CVD) and stroke (7). With this strong evidence, the World Health Organization specified a global target of reducing the daily sodium intake in adults to < 2 g (8). The American Heart Association recommends a sodium intake of up to 2,300 mg/day and transitioning to an ideal limit of up to 1,500 mg/day in adults (9), which was challenged in several studies that showed a non-significant (10–12), inverse (13, 14), or U- or J-shaped association (15, 16) between sodium intake and all-cause or CVD mortality.

Given its importance in cellular functions and antioxidant properties, a relatively high potassium intake of 4.7 g/day is recommended in healthy adults (17) and is associated with a lower risk of all-cause or CVD mortality in the general population (18–22), presumably due to its effect in improving BP, slowing the progression of chronic kidney disease (CKD), and decreasing CVD risk (23). Several epidemiologic studies examined the combined effects of sodium and potassium on the incidence or mortality from CVD (18, 21, 24), but a review showed no significant association between potassium intake and health or mortality outcomes, and this needs further research (25). The inconsistency in the abovementioned results may be attributable to genetic, environmental, behavioral, and dietary heterogeneity in various ethnic groups and different study design and chronic health conditions. Moreover, few studies have compared the effect of dietary sodium and potassium and the sodium–potassium ratio on mortality.

We aimed to examine the association between dietary sodium and potassium intake, sodium–potassium ratio, and all-cause and CVD mortality in the Korean general population by using large-scale cohort data. Furthermore, we investigated the abovementioned associations in patients with hypertension and CKD who were highly influenced by dietary sodium and potassium and examined which—sodium, potassium, and sodium–potassium ratio—conferred maximal accuracy in predicting mortality.

## Materials and methods

### Participants

**FIGURE 1:**
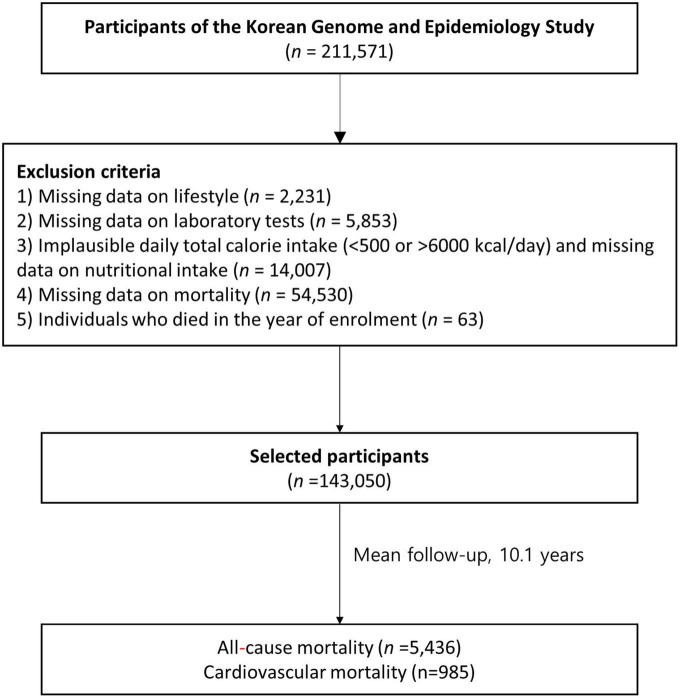
Flowchart of the participant selection process in this study.

We analyzed the baseline data of adult participants (age ≥ 40 years; Figure 1) from the Korean Genome and Epidemiology Study (KoGES)_Ansan-Ansung Study (2001–2002), KoGES_Health Examinee Study (2004–2013), and KoGES_Cardiovascular Disease Association Study (2005–2011)—large-scale, longitudinal, prospective cohort studies on the risk factors for non-communicable diseases—that were obtained from a previous study (26). Of the 211,571 participants in the baseline survey (2001–2013), we included 143,050 participants after excluding those lacking data on: (1) age and lifestyle factors (*n* = 2,231); (2) laboratory test results (*n* = 5,853); (3) dietary information and implausible total calorie intake (< 500 or > 6000 kcal/day; *n* = 14,007); and (4) mortality (*n* = 54,530) as well as (4) individuals who died in the year of enrolment (*n* = 63). The KoGES study protocol was reviewed and approved by the institutional review board (IRB) of the Korea Centers for Disease Control and Prevention. Informed consents were obtained from all participants. This study was approved by the IRB of Yongin Severance Hospital (IRB number: 3-2020-0043).

### Dietary assessment

Dietary intakes were assessed using a semiquantitative 103-item food frequency questionnaire (FFQ), administered by a trained interviewer, that was developed for a community-based cohort of the KoGES and recorded the frequency of consumption of each food per participant in the past year. Well trained interviewers conducted a FFQ using the face-to-face interview method. Individuals’ nutrient intakes were calculated using the nutrient database of each food item, recipes, nutrients by recipes, food intake frequency, and portion size for each food item. Detailed method for nutrition assessment was described in the manual of Korean Genome and Epidemiology Study.^1^ Dietary Na and K intakes (mg/day) were divided into quintiles, and the dietary sodium-to-potassium ratio was calculated by dividing Na consumption by the K consumption.

### Covariates

All health examination procedures were performed by trained medical staff. Blood pressures were measured twice with participants in the seated position. Blood tests were conducted after 8-h fasting. Serum glucose, glycated hemoglobin (HbA1C), total cholesterol (TC), high-density lipoprotein (HDL), and triglyceride (TG) levels were enzymatically determined using a Chemistry Analyzer (Hitachi 7600, Tokyo, Japan until August 2002, and ADVIA 1650, Siemens, Tarrytown, NY from September 2002). Smoking (current, former, and never smoker), alcohol intake (current, former, and never drinker), and physical activity (regular exerciser was defined as one who regularly exercised until sweating) were self-reported in a questionnaire. Hypertension was defined based on SBP ≥ 140 mmHg, DBP ≥ 90 mmHg, or current treatment with antihypertensive medication. Diabetes was defined based on fasting plasma glucose level ≥ 126 mg/dL, HbA1C ≥ 6.5%; or current antidiabetic therapy. Dyslipidemia was defined as TC ≥ 200 mg/dL, TG ≥ 150 mg/dL, or current treatment with antidyslipidemic medications. Obesity was defined as body mass index ≥ 25 kg/m^2^. CKD was defined as an estimated glomerular filtration rate < 60 mL/min/1.73 m^2^.

### Study outcomes

Mortality status was ascertained through data linkage based on the unique personal identification key code system as the KoGES data are linked to national data sources (Korea National Statistical Office), including mortality records. Participant mortality was tracked until December 2019 and mortality causes were classified based on the International Classification of Diseases (ICD) codes listed in the National Mortality Index. All-cause mortality included all deaths of specified and unknown causes whereas CVD mortality includes deaths under ICD-10 codes I00–I99.

### Statistical analysis

Data are presented as the mean (SD) and number (percentage). Dietary intakes of Na and K and the Na/K ratio were categorized into quintiles (1st quintile as reference). The incidences of all-cause and CVD mortality over the follow-up period were considered a dichotomous variable. The baseline characteristics of participants stratified by mortality status were compared using the independent *t*-test and chi-square test for continuous and categorical variables, respectively. Cox proportional hazards regression models with person-years as the time metric, were used to estimate hazard ratios (HRs) and 95% confidence intervals (CIs) of the association between dietary Na and K and their ratio with regard to the outcomes. Variables found to be closely associated with mortality (*P* < 0.05) were imported into the final multivariable models, as were variables known to be previously reported mortality-associated factors including age, sex, BMI, alcohol intake, smoking, regular exercise, total calorie intake, dyslipidemia, hypertension, diabetes and CKD (27, 28).

Time to events for all-cause and CVD mortality were defined as time to the end of follow-up (censored cases), wherein participants were censored at the time of all-cause or CVD mortality or at the end of study follow-up (December 2019). Kaplan–Meier curves with the log-rank test were used to compare cumulative rates of incident all-cause or CVD mortality according to the quintiles of the daily Na and K intake and Na/K ratio. To evaluate the predictability of the sodium, potassium, and sodium-to-potassium intake ratio, we calculated Harrell’s c-index (95% CI). All statistical analyses were performed using SAS 9.2 (SAS Institute, Cary, NC). Two-sided *p*-values < 0.05 were considered statistically significant.

## Results

Among the 143,050 participants, of whom 34.6% were male, the mean ± *SD* age was 53.5 ± 8.5 years. During the 10-year follow-up period, 5,436 all-cause and 985 CVD mortalities occurred. The mean ± *SD* sodium intake, potassium intake, and sodium-to-potassium ratio of the study cohort was 2,511.2 ± 1,424.7 mg/day, 2,216.5 ± 1,049.8 mg/day, and 1.14 ± 0.40, respectively, and the mortality-stratified baseline characteristics are summarized in Table 1. All-cause mortality showed a higher prevalence in participants who were male and older; with higher WC, SBP, DBP, glucose, HbA1C, and TG; current smokers; and those with hypertension, diabetes, and CKD, but showed a lower prevalence in those with lower BMI, TC, and HDL-C; in never drinkers and regular exercisers. Participants with all-cause mortality events consumed fewer total calories (kcal/day), Na (mg/day), and K (mg/day), and had a higher sodium-to-potassium ratio. Participants with CVD mortality were more likely to be men, older; have higher WC, SBP, DBP, glucose, HbA1C, TG; and lower TC and HDL-C; and CVD mortality showed a higher prevalence among current smokers and those with hypertension, diabetes, obesity and CKD, but a lower prevalence among never drinkers and regular exercisers. Participants with CVD mortality consumed fewer total calories (kcal/day), Na (mg/day), and K (mg/day) and had a higher sodium-to-potassium ratio.

**TABLE 1:** Baseline characteristics of study population based on mortality status.

Variables	Alive	Total death	CVD death	P1	P2
*n*	137,614	5,436	985		
Sex (men)	47,640 (34.6)	3,304 (60.8)	558 (56.65)	< 0.001	<0.001
Age (years)	53.5 ± 8.5	62.8 ± 9.3	64.9 ± 9.2	< 0.001	<0.001
BMI (kg/m^2^)	24.0 ± 2.9	23.9 ± 3.2	24.1 ± 3.3	0.03	0.31
Obesity, *n* (%)	46,236 (33.6)	1,858 (34.2)	370 (37.6)	0.374	0.009
WC (cm)	81.2 ± 8.6	84.1 ± 9.0	84.8 ± 9.2	< 0.001	<0.001
SBP (mmHg)	122.5 ± 15.3	127.2 ± 17.2	130.6 ± 18.1	< 0.001	<0.001
DBP (mmHg)	76.2 ± 10.0	77.5 ± 10.4	78.8 ± 10.9	< 0.001	<0.001
Glucose (mg/dL)	95.3 ± 20.6	104.3 ± 34.5	105.0 ± 34.8	< 0.001	<0.001
HbA1c (%)	5.7 ± 0.7	6.0 ± 1.2	6.1 ± 1.1	< 0.001	<0.001
TC (mg/dL)	197.6 ± 35.5	191.3 ± 39.0	194.8 ± 39.0	< 0.001	0.03
HDL-C (mg/dL)	52.8 ± 13.0	48.4 ± 13.2	47.0 ± 11.7	< 0.001	<0.001
LDL-C (mg/dL)	119.2 ± 32.6	114.4 ± 35.3	118.2 ± 36.1	< 0.001	0.36
Triglycerides (mg/dL)	128.9 ± 90.1	144.1 ± 107.5	148.9 ± 94.9	< 0.001	<0.001
Smoking status, n (%)				< 0.001	<0.001
Never smoker	100,083 (72.7)	2,704 (49.7)	515 (52.3)		
Former smoker	20,476 (14.9)	1,409 (25.9)	225 (22.8)		
Current smoker	17,055 (12.4)	1,323 (24.3)	245 (24.9)		
Alcohol intake, n (%)				< 0.001	<0.001
Never drinker	70,007 (50.9)	2,365 (43.5)	458 (46.5)		
Former drinker	5,221 (3.8)	564 (10.4)	88 (8.9)		
Current drinker	62,386 (45.3)	2,507 (46.1)	439 (44.6)		
Regular exercise (No)	67,974 (49.4)	3,347 (61.6)	658 (66.9)	< 0.001	<0.001
Hypertension, n (%)	22,996 (16.7)	1,411 (26.0)	324 (32.9)	< 0.001	<0.001
Diabetes, n (%)	9,526 (6.9)	838 (15.4)	153 (15.5)	< 0.001	<0.001
Dyslipidemia,, n (%)	78,211 (56.8)	3,008 (55.3)	591 (60.0)	0.03	0.05
CKD, *n* (%)	3,290 (2.4)	602 (11.1)	149 (15.1)	< 0.001	<0.001
Residential area, n (%)				< 0.001	<0.001
Urban	120,808 (87.8)	3,548 (65.3)	565 (57.4)		
Rural	16,806 (12.2)	1,888 (34.7)	420 (42.6)		
Total energy (kcal/day)	1,739.6 ± 542.0	1,637.2 ± 531.7	1,607.5 ± 558.6	< 0.001	<0.001
Carbohydrate (g/day)	310.1 ± 89.8	297.7 ± 87.0	293.6 ± 88.4	< 0.001	<0.001
Carbohydrate (%)	71.9 ± 7.0	73.6 ± 7.1	74.2 ± 7.6	< 0.001	<0.001
Fat (g/day)	27.5 ± 17.2	23.6 ± 16.9	22.7 ± 19.4	< 0.001	<0.001
Fat (%)	13.7 ± 5.5	12.2 ± 5.6	11.8 ± 5.9	< 0.001	<0.001
Protein (g/day)	58.7 ± 24.8	53.3 ± 24.9	51.7 ± 28.4	< 0.001	<0.001
Protein (%)	13.3 ± 2.6	12.8 ± 2.6	12.5 ± 2.7	< 0.001	<0.001
Sodium (mg/day)	2,513.2 ± 1,422.4	2,460.1 ± 1,479.1	2,359.8 ± 1,364.3	0.01	0.001
Potassium (mg/day)	2,225.7 ± 1,049.3	1,983.6 ± 1,035.7	1,877.4 ± 1,011.9	< 0.001	<0.001
Sodium/Potassium	1.14 ± 0.39	1.26 ± 0.46	1.29 ± 0.47	< 0.001	<0.001

BMI, Body mass index; CKD, Chronic kidney disease; DBP, Diastolic blood pressure; HDL-C, High density lipoprotein cholesterol; LDL-C, Low density lipoprotein cholesterol; SBP, Systolic blood pressure; TC, Total cholesterol; WC, Waist circumference.

Data are presented as mean ± standard deviations or number (%).

*P*-values were derived from independent *t*-test for continuous variables and chi-square test for categorical variables; significance was set at *p* < 0.05. P1: Alive vs. total death, P2: Alive vs. CVD death.

Percent of energy intake from carbohydrate or protein were calculated as follows: carbohydrate intake (g/day) or protein intake (g/day)/total energy intake (kcal/day) × 100%. The percent of energy intake from fat was calculated as follows: fat intake (g/day) × 9 kcal/total energy intake (kcal/day) × 100%.

**FIGURE 2:**
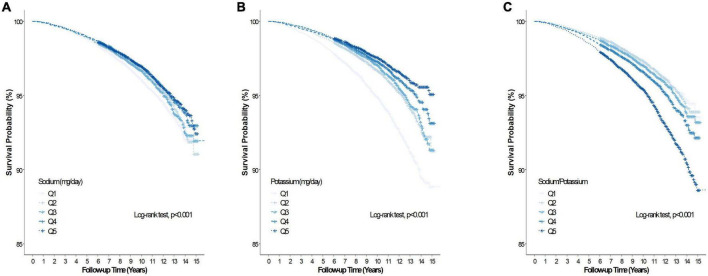
Kaplan–Meier curve of the association between salt intake and all-cause. **(A)** Association between dietary sodium (Na) and all-cause mortality. **(B)** Association between dietary potassium (K) and all-cause mortality. **(C)** Association between dietary sodium-to-potassium (Na/K) ratio and all-cause mortality.

**FIGURE 3:**
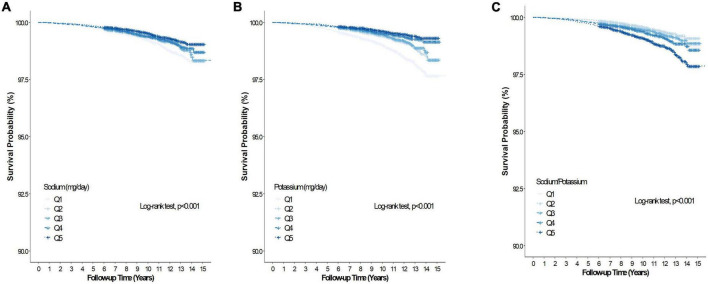
Kaplan–Meier curve of the association between salt intake and cardiovascular disease mortality. **(A)** Association between dietary sodium and cardiovascular disease mortality. **(B)** Association between dietary potassium and cardiovascular disease mortality. **(C)** Association between dietary Na/K ratio and cardiovascular disease mortality.

During the mean (range) follow-up of 10.1 (0.2–15.9) years, 5,436 and 985 participants had all-cause and CVD deaths, respectively. Table 2 summarizes the association between baseline Na intake, K intake, sodium-to-potassium ratio and all-cause and CVD mortality of the study cohort. In the unadjusted model, Na intake was inversely associated with all-cause mortality (Q5 vs. Q1, HR 0.79, 95% CI 0.73–0.85), but was unassociated with all-cause mortality after adjustment for age, sex, BMI, alcohol intake, smoking, regular exercise, total calorie intake, dyslipidemia, hypertension, diabetes, CKD, and K intake (Q5 vs. Q1, HR 1.09, 95% CI 0.97–1.22). Sodium intake was inversely associated with CVD mortality (Q5 vs. Q1, HR 0.60, 95% CI 0.49–0.73) in the unadjusted model, but not after adjustment for the abovementioned confounding factors (Q5 vs. Q1, HR 0.92, 95% CI 0.70–1.21). Figures 2A, 3A presents the Kaplan–Meier curves from the log-rank test, and depicts a lower risk for cumulative all-cause and CVD mortality with Na intake in Q5, followed by Q4, Q3, Q2, and Q1 (both log rank *p* < 0.001). Potassium intake was significantly associated inversely with all-cause and CVD mortality (Q5 vs. Q1) in the unadjusted model (HR, 95% CI 0.46, 0.42–0.50 and 0.35, 0.29–0.43, respectively), and the trend persisted after adjusting for age, sex, BMI, alcohol intake, smoking, regular exercise, total calorie intake, dyslipidemia, hypertension, diabetes, CKD, and Na intake (HR, 95% CI, 0.79, 0.69–0.91 and 0.68, 0.48–0.95, respectively). Figures 2B, 3B present the Kaplan–Meier curves from the log-rank test, which show a lower risk for cumulative all-cause and CVD mortality with K intake in Q5, followed by Q4, Q3, Q2, and Q1 (both log rank *p* < 0.001). The sodium-to-potassium ratio was significantly associated with all-cause and CVD mortality in the unadjusted model (Q5 vs. Q1, HR, 95% CI 1.76, 1.62–1.91, and 2.00, 1.65–2.43, respectively), but the association disappeared after adjustment for age, sex, BMI, alcohol intake, smoking, regular exercise, total calorie intake, dyslipidemia, hypertension, diabetes, and CKD (Q5 vs. Q1, HR, 95% CI, 1.06, 0.97–1.15, and 1.12, 0.92–1.37, respectively). Figures 2C, 3C present the Kaplan–Meier curves from the log-rank test, showing a higher risk for cumulative all-cause and CVD mortality with the sodium-to-potassium ratio in Q5, followed by Q4, Q3, Q2, and Q1 (both log rank *p* < 0.001).

**TABLE 2:** Multiple Cox proportional hazard regression analysis for total death and CVD death according to sodium (Na), potassium (K) and sodium to potassium (Na/K) ratio intake.

Hazard ratios (95% Confidence intervals)					
**Na (mg/day)**	Q1 (19.7, 1,363.9)	Q2 (1,363.9, 1,989.0)	Q3 (1,989.0, 2,602.2)	Q4 (2,602.2, 3,422.7)	Q5 (3,422.7, 18,190.2)
**Total death**					
Unadjusted	1.00 (ref)	0.84 (0.78–0.91)	0.89 (0.82–0.96)	0.80 (0.073–0.87)	0.79 (0.73–0.85)
Model 1	1.00 (ref)	0.92 (0.85–1.00)	0.98 (0.90–1.06)	0.92 (0.81–1.00)	0.95 (0.87–1.05)
Model 2	1.00 (ref)	0.93 (0.86–1.01)	0.98 (0.90–1.07)	0.93 (0.85–1.01)	0.96 (0.87, 1.05)
Model 3*	1.00 (ref)	1.00 (0.91–1.09)	1.08 (0.99–1.18)	1.05 (0.95–1.10)	1.09 (0.97–1.22)
**CVD death**					
Unadjusted	1.00 (ref)	0.66 (0.54–0.80)	0.79 (0.66–0.95)	0.75 (0.62–0.90)	0.60 (0.49–0.73)
Model 1	1.00 (ref)	0.74 (0.61–0.90)	0.92 (0.76–1.11)	0.89 (0.74–1.09)	0.75 (0.60–0.94)
Model 2	1.00 (ref)	0.75 (0.62–0.91)	0.92 (0.76–1.11)	0.90 (0.74–1.10)	0.75 (0.60–0.94)
Model 3*	1.00 (ref)	0.79 (0.65–0.98)	1.01 (0.82–1.24)	1.03 (0.82–1.30)	0.92 (0.70–1.21)
**K (mg/day)**	Q1 (130.4, 1,405.4)	Q2 (1405.4, 1,830.6)	Q3 (1,830.7, 2,267.7)	Q4 (2,267.7, 2,884.4)	Q5 (2,844.5, 19,268.3
**Total death**					
Unadjusted	1.00 (ref)	0.65 (0.60–0.70)	0.60 (0.55–0.65)	0.53 (0.49–0.57)	0.46 (0.42–0.50)
Model 1	1.00 (ref)	0.82 (0.76–0.89)	0.86 (0.79–0.93)	0.83 (0.76–0.91)	0.84 (0.76–0.94)
Model 2	1.00 (ref)	0.83 (0.77–0.90)	0.86 (0.79–0.93)	0.84 (0.76–0.92)	0.85 (0.76–0.95)
Model 3†	1.00 (ref)	0.81 (0.75–0.89)	0.76 (0.75–0.91)	0.80 (0.71–0.89)	0.79 (0.69–0.91)
**CVD death**					
Unadjusted	1.00 (ref)	0.60 (0.50–0.72)	0.55 (0.46–0.66)	0.43 (0.35–0.52)	0.35 (0.29–.43)
Model 1	1.00 (ref)	0.81 (0.68–0.97)	0.86 (0.71–1.05)	0.73 (0.58–0.91)	0.67 (0.51–0.88)
Model 2	1.00 (ref)	0.82 (0.69–0.99)	0.87 (0.71–1.06)	0.74 (0.59–0.92)	0.68 (0.51–0.89)
Model 3†	1.00 (ref)	0.83 (0.68–1.01)	0.86 (0.68–1.08)	0.72 (0.55–0.95)	0.68 (0.48–0.95)
**Na/K**	Q1 (0.08, 0.80)	Q2 (0.80, 1.00)	Q3 (1.00, 1.20)	Q4 (1.20, 1.45)	Q5 (1.45, 4.18)
**Total death**					
Unadjusted	1.00 (ref)	0.99 (0.90–1.08)	1.09 (0.99–1.20)	1.25 (1.15–1.37)	1.76 (1.62–1.91)
Model 1	1.00 (ref)	0.98 (0.89–1.08)	1.02 (0.93–1.12)	1.01 (0.92–1.10)	1.06 (0.97–1.15)
Model 2	1.00 (ref)	0.98 (0.89–1.08)	1.02 (0.93–1.12)	1.01 (0.92–1.10)	1.06 (0.97–1.15)
**CVD death**					
Unadjusted	1.00 (ref)	0.92 (0.73–1.16)	0.15 (0.92–1.44)	1.37 (1.11–1.69)	2.00 (1.65–2.43)
Model 1	1.00 (ref)	0.92 (0.73–1.17)	1.09 (0.88–1.37)	1.10 (0.89–1.36)	1.13 (0.93–1.38)
Model 2	1.00 (ref)	0.93 (0.73–1.17)	1.09 (0.88–1.36)	1.10 (0.89–1.36)	1.12 (0.92–1.37)

Model 1: adjusted for age, sex, BMI, alcohol intake, smoking, regular exercise, and total calorie intake. Model 2: adjusted for age, sex, BMI, alcohol intake, smoking, regular exercise, total calorie intake, dyslipidemia, hypertension, diabetes and CKD; Model 3*: Model 2 + potassium intake; Model 3†: Model 2 + sodium intake.

Furthermore, using the c-index, we compared the predictive power of dietary Na, K, and the sodium-to-potassium ratio for all-cause mortality. The c-index of dietary K intake was significantly higher than that of the Na intake and the sodium-to-potassium ratio (K vs. Na, *p* < 0.001; K vs. Na/K ratio, *p* = 0.021; data not shown). In the subgroup analysis of the association between the dietary Na, K, and sodium-to-potassium ratio and all-cause mortality in patients with hypertension and CKD (Supplementary Figure 1), the mean ± *SD* Na intake, K intake, and sodium-to-potassium ratio in patients with hypertension and in those with CKD were 2,578.6 ± 1,491.6 mg/day, 2,178.3 ± 1,051.7 mg/day, and 1.19 ± 0.42 and 2,300.8 ± 1,349.9 mg/day, 1,946.0 ± 965.6 mg/day, and 1.20 ± 0.45, respectively. After adjusting for confounders, only K intake was significantly associated inversely with all-cause mortality in patients with hypertension and CKD. However, no significant associations were detected for CVD mortality and dietary Na and K intake, which might be attributable to the small number of CVD events (data not shown).

## Discussion

This study revealed a significant inverse association between K intake and all-cause and CVD mortality during a mean follow-up duration of 10.1 years in a large population-based Korean cohort. However, dietary Na intake and the sodium-to-potassium ratio were unassociated with mortality, even in the subgroup of patients with hypertension and CKD. Furthermore, compared to Na intake and the sodium-to-potassium ratio, we found that K intake was the most significant predictive factor for all-cause and CVD mortality.

Sodium intake is crucial for maintaining extracellular fluid balance and life processes (29), and is regulated within a physiological range by renal, neural, and hormonal processes (30). Similarly, K fulfills various physiological roles such as determining the resting membrane potential and plasma volume (2). However, excess Na intake induces increased arterial stiffness (31), elevated blood pressure (4–6), left ventricular hypertrophy (32), and worse renal function (33), whereas high dietary K intake can counteract the abovementioned effects by activating nitric oxide release and decreasing arterial stiffness (34). Therefore, the dietary combination of lower Na and higher K intake has been recommended as a strategy to prevent hypertension, CKD, and CVD (35). Evidence from numerous epidemiologic and clinical studies supports the theory that inadequate intake of Na and K is likely associated with increased cardiovascular morbidity and mortality (4–6, 19, 21). Moreover, several studies have shown that the combined effect of a sodium and potassium ratio on CVD is stronger than that of either Na or K alone (18, 24, 36).

Nonetheless, there are inconsistent findings about the association between Na intake and mortality (10–16). Cohen et al. found that high Na intake, which was assessed by the 24-h recall method, was either inversely associated or was unlikely to be independently associated with increased risk of all-cause and CVD mortality (12, 14). The Health, Aging, and Body Composition (Health ABC) Study revealed that Na intake, assessed by the FFQ, was unassociated with 10-year mortality in older adults (11). Recently, Messerli et al. reported data from multiple countries on an inverse association between dietary Na intake and mortality (13), which suggested the need for caution when recommending the current guideline for low Na intake without strong evidences. Strict Na control may have an unfavorable effect on insulin resistance (37), the serum lipid level (38), and neurohormonal activity (38) that could predispose to CVD and heart failure. Therefore, the 2013 Institute of Medicine (IOM) reported that there was no evidence for suggesting that a risk of adverse health outcomes was associated with Na intake levels of 1,500–2,300 mg/day (39).

In line with previous studies, we observed an insignificant association between Na intake and all-cause and CVD mortality. Several possible mechanisms could explain our findings. First, the sources of Na intake could differ by race, region, and country (40). Sodium that was added during food manufacturing and processing was the leading contributor of Na intake in Western countries (41). However, the traditional Korean diet is characterized by high consumption of grains, fermented foods (kimchi, soy sauce, and soybean paste), vegetables, legumes, and fish that are seasoned with garlic, green onion, red pepper, and ginger and has a high Na content. O’Donnell et al. (42) suggested that some high-Na food items (e.g., vegetables, legumes, and fish) are beneficial for the overall health. Similarly, the Japanese dietary pattern, which predominantly contains soybean products, fish, seaweed, vegetables, and fruits, was inversely associated with CVD mortality despite the high Na content (43). Second, there might be differences in salt sensitivity between Asian and Western populations (44). Given the enhanced Na sensitivity in obese individuals, in the Korean population, which has a relatively low BMI compared to the Western population, the effect of Na on BP could be weaker (45). Third, the mean Na intake in this study was approximately 2.5 mg/day which corresponds to the range of Na intake (2.3–4.6 g/day) that is unassociated with an increased risk of CVD and mortality (46, 47).

Several studies have shown the beneficial effects of K intake from plant-based foods on CVD and mortality in the general population (4–6, 19, 21, 34), although the effects of K intake on health remains debatable (25, 48, 49). The current recommendation by the IOM indicates that a K intake of 4.7 g/day is adequate (3). The participants in our study consumed approximately half of the recommended amount of K, and K intake was significantly associated with a lower risk of all-cause and CVD mortality. Moreover, the c-index of dietary K intake was significantly higher than that of Na intake and the sodium-to-potassium ratio, suggesting that K intake is the most influential dietary factor for mortality. Potassium-rich foods have anti-inflammatory and antioxidant factors derived from legumes, whole grains, vegetables, and fruits (2), and low K intake might reflect poor diet quality (20). Interestingly, in a recent prospective study, lower dietary K intake was associated with a higher mortality risk in hemodialysis patients, who are usually guided by dietary K restriction to prevent potential hyperkalemia (50). In our study, the sodium-to-potassium ratio was unassociated with all-cause and CVD mortality. Although the exact mechanisms remain elusive, the non-significant relationship between Na intake and mortality could attenuate the association between the sodium-to-potassium ratio and mortality.

Our study has several limitations. First, we assessed dietary Na and K intake using the only the FFQ, which, although widely used in large-scale population-based studies, confers a risk of recall bias, variations in the Na content of food items, and lack of information on the addition of table salt (42). Moreover, dietary nutrient intake could be underestimated. Second, we could not obtain the data of 24-h urine collection, which is an accurate measure for estimating Na and K intake (51). However, 24-h urine collection is burdensome for participants and proves impractical in large-scale epidemiological studies. Third, we only assessed the baseline Na and K intake, and these intakes were not updated during the follow-up period. Thus, baseline exposures might not reflect changes in Na and K intakes over time. Forth, since we included participants with comorbidities, there could be undisclosed general medical conditions and other residual factors that we did not adequately take into account. Finally, we included middle-aged and older Korean adults, which limits the generalizability of our results to other countries and ethnic groups.

Nonetheless, our study has several strengths. We analyzed large-scale population-based data and ascertained all-cause and CVD mortality over a long follow-up. This is the first study to compare the effect of dietary Na and K and the sodium-to-potassium ratio on mortality. Our results are supported by subgroup analyses in patients with hypertension and CKD who were highly influenced by dietary Na and K.

## Conclusion

Potassium intake was inversely associated with all-cause and CVD mortality. Sodium intake and the sodium-to-potassium ratio were unassociated with all-cause and CVD mortality in a large Korean population. From a public health perspective, a healthy dietary pattern that incorporates sufficient K-rich plant-based foods should be emphasized. However, large-scale randomized clinical trials are needed to validate the results of this study.

## Data availability statement

Publicly available datasets were analyzed in this study. This data can be found here: https://www.kdca.go.kr/contents.es?mid=a40504010000.

## Ethics statement

This study was approved by the IRB of Yongin Severance Hospital (IRB number: 3-2020-0043). The patients/participants provided their written informed consent to participate in this study.

## Author contributions

Y-JK, HL, GP, and J-WL contributed to the conception, design of the work, acquisition, analysis, interpretation of the data, and drafting of the manuscript. All authors critically revised the manuscript, provided final approval, and agreed to be accountable for all aspects of the work, while ensuring integrity and accuracy.

## Footnotes

1
https://www.kdca.go.kr/contents.es?mid=a40504010000
