# Supplementary material for: Association between dietary sodium, potassium, and the sodium-to-potassium ratio and mortality: A 10-year analysis
Source: Front Nutr. 2022 Nov 11;9:1053585. doi: 10.3389/fnut.2022.1053585 (PMC9691953; doi:10.3389/fnut.2022.1053585)
Supplement: Supplementary file 1 [file Data_Sheet_1.PDF]

Supporting information

Title: Association between dietary sodium, potassium, and the sodium-to-potassium ratio and mortality: A 10-year analysis

Number of eFigures:1

eFigure 1. Subgroup analysis for association between dietary sodium (Na), potassium (K) or sodium to potassium (Na/K) ratio intake with total death in patients with hypertension or CKD

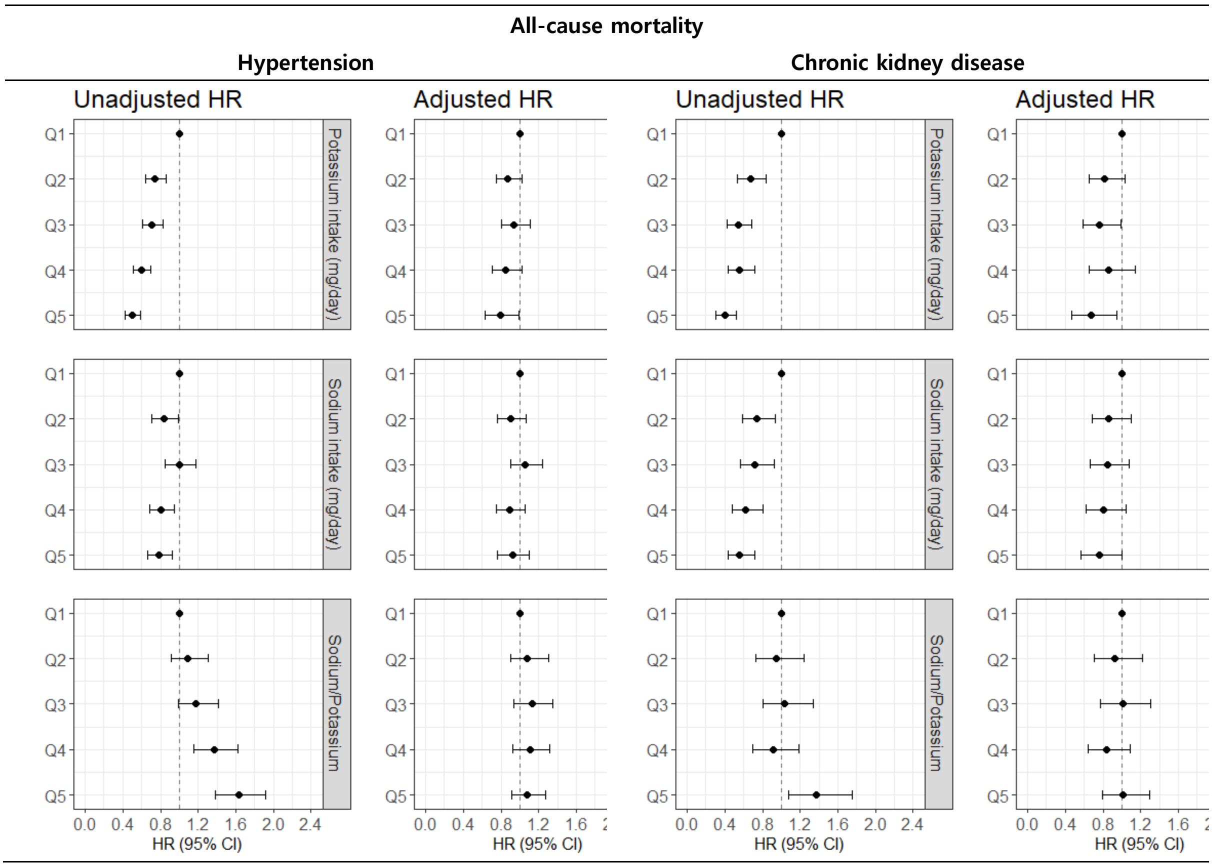

Patients with Hypertension					
		Hazard ratios (95% Confidence intervals)			
Na (mg/day)	Q1 (19.6, 1369.9)	Q2 (1370.0, 2039.9)	Q3 (2040.0, 2661.3)	Q4 (2661.3, 3522.8)	Q5 (3522.9, 15236.6)
Unadjusted	1.00 (ref)	0.84 (0.71–0.99)	1.00 (0.85–1.17)	0.81 (0.69–0.95)	0.78 (0.66–0.92)
Adjusted	1.00 (ref)	0.90 (0.76–1.07)	1.06 (0.90–1.24)	0.89 (0.75–1.06)	0.92 (0.76–1.10)
K (mg/day)	Q1 (130.4, 1359.6)	Q2 (1359.7, 1786.1)	Q3 (1786.1, 2224.7)	Q4 (2225.2, 2849.4)	Q5 (2849.5, 12750.8)
Unadjusted	1.00 (ref)	0.74 (0.64–0.86)	0.71 (0.61–0.83)	0.60 (0.51–0.70)	0.50 (0.42–0.59)
Adjusted	1.00 (ref)	0.87 (0.75–1.02)	0.94 (0.80-1.11)	0.85 (0.71–1.02)	0.79 (0.63–0.99)
Na/K	Q1 (0.09, 0.83)	Q2 (0.83, 1.05)	Q3 (1.05, 1.26)	Q4 (1.26, 1.52)	Q5 (1.52, 3.61)
Unadjusted	1.00 (ref)	1.09 (0.91–1.31)	1.18 (0.99–1.42)	1.37 (1.15–1.62)	1.63 (1.38–1.92)
Adjusted	1.00 (ref)	1.08 (0.90–1.31)	1.13 (0.94–1.35)	1.11 (0.93–1.32)	1.08 (0.91–1.27)
**Patients with CKD**					
		Hazard ratios (95% Confidence intervals)			
Na (mg/day)	Q1 (23.2, 1190.8)	Q2 (1191.2, 1782.0)	Q3 (1782.3, 2391.2)	Q4 (2391.9, 3200.5)	Q5 (3202.5, 12551.7)
Unadjusted	1.00 (ref)	0.74 (0.59–0.94)	0.72 (0.57–0.92)	0.62 (0.48–0.80)	0.56 (0.44–0.72)
Adjusted	1.00 (ref)	0.86 (0.68-1.10)	0.85 (0.66-1.08)	0.80 (0.62–1.05)	0.76 (0.57–1.00)
K (mg/day)	Q1 (178.2, 1158.4)	Q2 (1158.7, 1577.2)	Q3 (1577.3, 2014.0)	Q4 (2014.0, 2628.6)	Q5 (2628.7, 10159.9)
Unadjusted	1.00 (ref)	0.67 (0.53–0.84)	0.54 (0.42–0.69)	0.56 (0.44–0.72)	0.40 (0.30–0.52)
Adjusted	1.00 (ref)	0.82 (0.65-1.03)	0.76 (0.59–0.99)	0.86 (0.65–1.14)	0.67 (0.47–0.95)
Na/K	Q1 (0.13, 0.81)	Q2 (0.81, 1.04)	Q3 (1.04, 1.27)	Q4 (1.27, 1.56)	Q5 (1.56, 3.61)
Unadjusted	1.00 (ref)	0.95 (0.73–1.24)	1.03 (0.80–1.34)	0.91 (0.70–1.19)	1.37 (1.08-1.75)
Adjusted	1.00 (ref)	0.93 (0.71–1.22)	1.01 (0.77–1.31)	0.84 (0.64–1.09)	1.01 (0.79-1.29)

Adjusted for age, sex, BMI, alcohol intake, smoking, regular exercise, and total calorie intake
